# Association between phospholipid metabolism in plasma and spontaneous preterm birth: a discovery lipidomic analysis in the cork pregnancy cohort

**DOI:** 10.1007/s11306-020-1639-6

**Published:** 2020-01-24

**Authors:** Aude-Claire Morillon, Shirish Yakkundi, Gregoire Thomas, Lee A. Gethings, James I. Langridge, Philip N. Baker, Louise C. Kenny, Jane A. English, Fergus P. McCarthy

**Affiliations:** 1 Department of Obstetrics and Gynaecology, The Irish Centre for Maternal and Child Health Research (INFANT), University College Cork, Cork, Ireland; 2 SQU4RE, 8800 Roeselare, Belgium; 3 Waters Corporation, Wilmslow, UK; 4 Division of Infection and Respiratory Medicine, Faculty of Biology, Medicine and Health, Manchester Institute of Biotechnology, University of Manchester, Manchester, UK; 5 College of Life Sciences, University of Leicester, Leicester, UK; 6 Department of Women’s and Children’s Health, Institute of Translational Medicine, University of Liverpool, Liverpool, UK; 7 Department of Anatomy and Neuroscience, University College Cork, Western Gateway Building, Western Road, Cork, Ireland

**Keywords:** Spontaneous preterm birth, Pregnancy, Metabolic profiling, Lipid profiling, Lipidomics, SCOPE study

## Abstract

**Introduction:**

Preterm birth (PTB) is defined as birth occurring before 37 weeks’ gestation, affects 5–9% of all pregnancies in developed countries, and is the leading cause of perinatal mortality. Spontaneous preterm birth (sPTB) accounts for 31–50% of all PTB, but the underlying pathophysiology is poorly understood.

**Objective:**

This study aimed to decipher the lipidomics pathways involved in pathophysiology of sPTB.

**Methods:**

Blood samples were taken from SCreening fOr Pregnancy Endpoints (SCOPE), an international study that recruited 5628 nulliparous women, with a singleton low-risk pregnancy. Our analysis focused on plasma from SCOPE in Cork. Discovery profiling of the samples was undertaken using liquid chromatography-mass spectrometry Lipidomics, and features significantly altered between sPTB (n = 16) and Control (n = 32) groups were identified using empirical Bayes testing, adjusting for multiple comparisons.

**Results:**

Twenty-six lipids showed lower levels in plasma of sPTB compared to controls (adjusted p < 0.05), including 20 glycerophospholipids (12 phosphatidylcholines, 7 phosphatidylethanolamines, 1 phosphatidylinositol) and 6 sphingolipids (2 ceramides and 4 sphingomyelines). In addition, a diaglyceride, DG (34:4), was detected in higher levels in sPTB compared to controls.

**Conclusions:**

We report reduced levels of plasma phospholipids in sPTB. Phospholipid integrity is linked to biological membrane stability and inflammation, while storage and breakdown of lipids have previously been implicated in pregnancy complications. The contribution of phospholipids to sPTB as a cause or effect is still unclear; however, our results of differential plasma phospholipid expression represent another step in advancing our understanding of the aetiology of sPTB. Further work is needed to validate these findings in independent pregnancy cohorts.

**Electronic supplementary material:**

The online version of this article (10.1007/s11306-020-1639-6) contains supplementary material, which is available to authorized users.

## Introduction

Preterm birth (PTB) is defined as birth occurring before 37 weeks of gestation and can be spontaneous or iatrogenic. Preterm delivery rates vary between countries, but are estimated to be 5–9% of pregnancies in Europe and other developed countries, with rates still rising in most industrialised countries (Goldenberg et al. 2008; Slattery and Morrison 2002). PTB accounts for 75% of perinatal deaths, with two-thirds of these deaths occurring in preterm infants delivered before 32 weeks of gestation (Slattery and Morrison 2002). Spontaneous preterm birth (sPTB) is estimated to occur in 31–50% of all PTB (Slattery and Morrison 2002) and the pathways involved are not yet fully understood. A previous study of low-risk nulliparous pregnant women in an international cohort, SCreening fOr Pregnancy Endpoints (SCOPE) study (www.scopestudy.net), used clinical data to determine risk factors linked to two sub-phenotypes of sPTB, sPTB with intact membranes (sPTB-IM) and sPTB occurring after prelabour rupture of membranes (sPTB-PPROM). Characteristics such as marijuana use before conception, shorter cervical length, or strong family history of low birth weight, were linked to higher risks of sPTB. However, the models built using the reported risk factors were not robust enough to predict sPTB (Dekker et al. 2012). Indeed, the area under the curve (AUC) of the model for sPTB-IM was 0.69, with a 0.39 sensitivity based on 90% specificity; the model for sPTB-PPROM showed an AUC of 0.79, with 0.49 sensitivity based on 90% specificity. As a result, research efforts have focused on finding blood based biomarkers for early diagnosis and intervention.

Metabolites are intermediate compounds involved in biochemical processes and are a reflection of cellular metabolic pathways of the organism. Similarly, lipids are a subset of metabolites which form the matrix of our cell membranes and support a variety of biological functions such as energy storage, cell metabolism, and cell signalling. Together, metabolites and lipids levels are regulated in response to environmental modifications (Goodacre et al. 2004), and as such, are a direct reflection of the phenotype (Patti et al. 2012); this makes them ideal candidates for biomarkers and therapeutic targets. Several recent studies have used metabolomics methods for biomarker discovery of PTB in plasma (Lizewska et al. 2018), in amniotic fluid samples (Menon et al. 2014; Romero et al. 2010), and in cervicovaginal secretions (Auray-Blais et al. 2011). A systematic review performed on the literature between 1965 and 2008 showed that various sample types have been used for the study of biomarkers of spontaneous preterm birth (Menon et al. 2011), and 46.2% of included studies used maternal blood samples, 31.0% used amniotic fluid samples, and 1.2% used urine samples. In addition, the collection of blood sample is minimally invasive compared to the collection of amniotic fluid for example, which can present risks for the fetus (Beta et al. 2018).

In recent years, several studies have linked altered levels of lipids and higher risk of adverse pregnancy outcomes (Baig et al. 2013; Jiang et al. 2017; Reece et al. 1997). Baig et al. have demonstrated that altered levels of glycerophospholipids and sphingolipids in syncytiotrophoblast microvesicles shed from the placenta, are associated with increased risk of preeclampsia or recurrent miscarriages (Baig et al. 2013). Reece et al. have shown an association between preterm birth (< 37 weeks of gestation) and higher levels of arachidonic acid and docosapentaneoic acid levels in maternal blood samples taken at the time of delivery (Reece et al. 1997). Jiang et al. preformed a systematic review aiming to analyse the associations between altered maternal lipids levels (cholesterol, triglycerides, high and low lipoprotein-cholesterol), and the risk of preterm birth (Jiang et al. 2017). Their meta-analysis showed that altered maternal levels of lipids are associated with a higher risk of preterm birth, for either higher levels [pooled OR for elevated levels 1.68 (95% CI 1.24–2.26)], or lower levels [pooled OR 1.52 (95% CI 1.13–3.82)].

To date, no study has identified metabolites or lipids robust enough to be used as biomarkers of PTB, as they need to be validated in different populations, using larger sample sizes. Here we describe the first discovery lipidomics study to be undertaken in sPTB. The aim of this discovery profiling study was to help determine the clinically relevant lipidomic pathways involved in the aetiology of sPTB. For this purpose, lipidomics profiles were obtained from plasma samples taken at 20 weeks of gestation from women recruited to the SCOPE study in Cork, Ireland (Kenny et al. 2014).

## Materials and methods

### Study participants

**Fig. 1:**
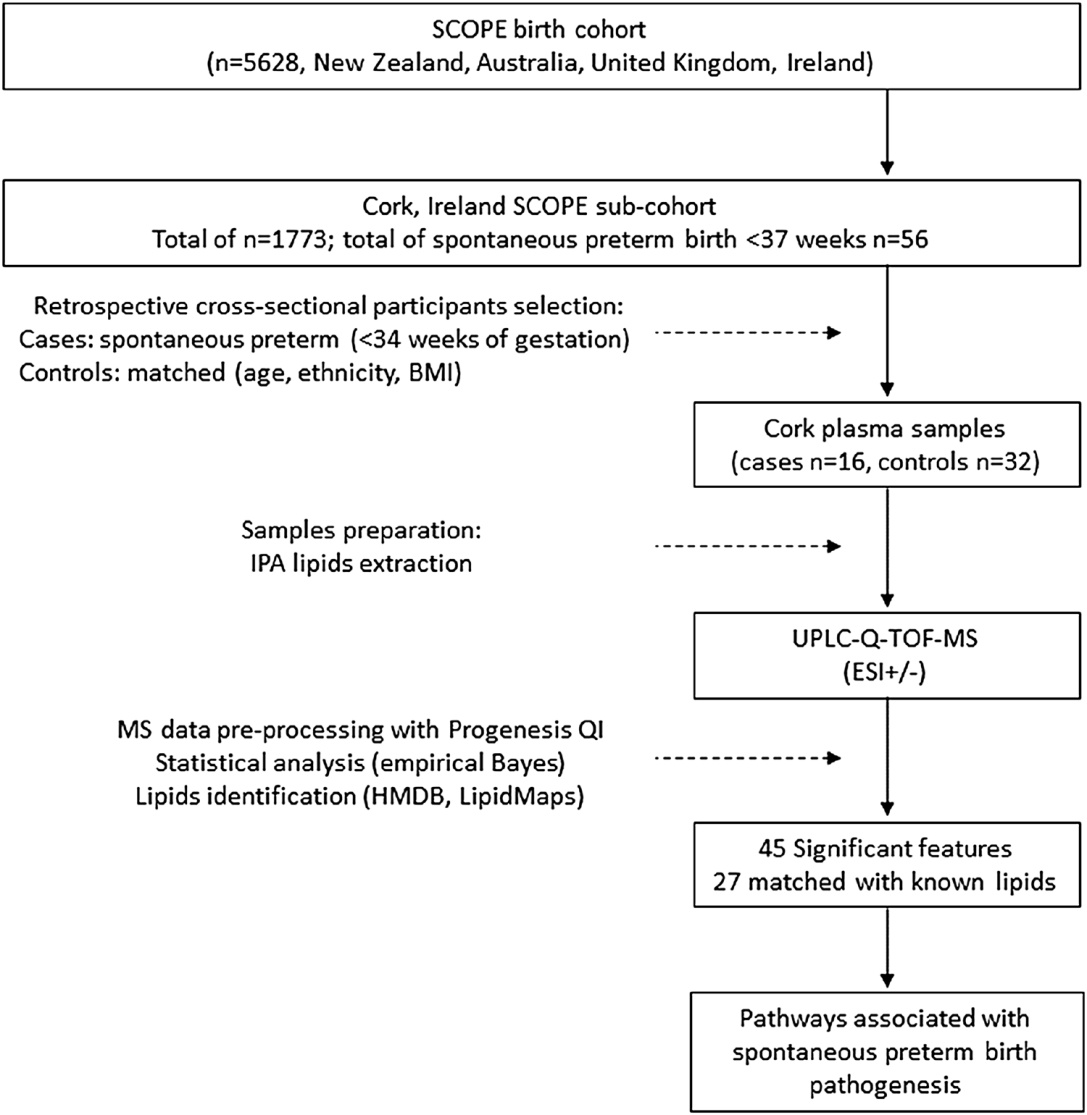
Flow chart of participant selection, samples preparation and analysis

Flow chart of participant selection and samples preparation are presented in Fig. 1. Selected participants were women recruited through the SCOPE study (www.scopestudy.net), in Cork, Ireland. SCOPE is an international longitudinal birth cohort that recruited over 5000 healthy nulliparous women with singleton pregnancies in Australia, New Zealand, the United Kingdom and Ireland (Kenny et al. 2014). Written informed consent and ethical approval were obtained prior to sample collection [ECM5 (10) 05/02/08]. In Cork SCOPE sub-cohort, 1773 women were recruited, including 56 women with spontaneous preterm birth before 37 weeks of gestation, which represents approximately 3% of this population. In the present cross-sectional study from a prospectively collected birth cohort, we chose to focus on women who had an “early” spontaneous preterm birth, defined as spontaneous preterm birth < 34 weeks of gestation (n = 16, 0.9% of Cork SCOPE population). Plasma samples were collected at 20 weeks of gestation, and women who later delivered prematurely before 34 weeks of gestation (cases) were matched to women who had an uncomplicated pregnancy (controls). Controls (n = 32) were matched to cases (n = 16) according to age (± 5 years), and body mass index (BMI, ± 3.0 kg/m^2^). Detailed clinical and demographic data is presented in Table 1.

**Table 1:** Demographic and clinical data of the study population

	Control (n = 32)	Case (n = 16)	p value
Age (years)	29.69 (5.03)	30.31 (6.93)	0.723
BMI, grouped according to World Health Organisation			
0– < 18.5, underweight	0	1 (6.2)	0.555
≥ 18.5 and < 25, normal weight	16 (50.0)	7 (43.8)	
≥ 25 and < 30, overweight	8 (25.0)	4 (25.0)	
≥ 30, obese	8 (25.0)	4 (25.0)	
Ethnicity			
Caucasian	32 (100)	16 (100)	NA
Asian	0	0	
Pacific Islander	0	0	
Maori	0	0	
Marital status			
Single	1 (3.1)	6 (37.5)	0.006
Married	21 (65.6)	7 (43.8)	
Stable relationship	10 (31.3)	3 (18.8)	
Years of schooling	14 (13–14)	13.5 (13–14)	0.801
Years of schooling, in groups			
< 12 years	0	2 (12.5)	0.120
12 or 13 years	15 (46.9)	6 (37.5)	
> 13 years	17 (53.1)	8 (50.0)	
Job situation			
Full time work	25 (78.1)	12 (75.0)	0.130
Part time work	4 (12.5)	0	
Homemaker	2 (6.3)	0	
Unemployed	1 (3.1)	2 (12.5)	
Student	0	1 (6.3)	
Sickness beneficiary	0	1 (6.3)	
Socioeconomic index	45.66 (19.02)	35.81 (13.31)	0.044
Smoking status at first visit (15 weeks)			
Never smoked	23 (71.9)	10 (62.5)	0.642
Smoked pre-pregnancy, but quit smoking before pregnant	0	0	
Smoked in pregnancy, but quit before first visit	6 (18.8)	3 (18.8)	
Smoking at first visit (15 weeks)	3 (9.4)	3 (18.8)	
Alcohol status at first visit (15 weeks)			
Never consumed alcohol	3 (9.4)	3 (18.8)	0.734
Consumed alcohol pre-pregnancy, but quit before conception	4 (12.5)	1 (6.3)	
Consumed alcohol in pregnancy, but quit before first visit	20 (62.5)	9 (56.3)	
Continuing to drink at first visit	5 (15.6)	3 (18.8)	
Birthweight (g)	3560 (3385–3775)	1665 (1102.5–2070)	< 0.001
Customised birthweight centile^a^	47.6 (34.18–81.1)	15.1 (9.03–73.85)	0.076
Gestational age at delivery (weeks)	40.50 (39.79–41.40)	32.29 (28.75–33.36)	< 0.001

Values are shown as mean (SD), median (interquartile range) or n (%)

*BMI* body mass index, *SGA* small for gestational age, *NA* not applicable; all mothers were nulliparous

^a^Customised birthweight centile: adjusted for mother’s height, weight at 15 weeks visit, ethnicity, sex and weight of baby and gestation at delivery of baby

### Reagents and materials

Liquid chromatography grade iso-propanol (IPA), acetonitrile (ACN) and ammonium formate were purchased from Fisher Scientific (Loughborough, UK). LC–MS glass vials and ultra-performance liquid chromatography (UPLC) columns were purchased from Waters (Waters, Wexford, Ireland).

### Sample preparation

Heparinised plasma samples taken from participants at 20 weeks’ gestation were randomised before extraction. Samples were taken out of – 80 °C storage and allowed to thaw on ice, before being transferred to labelled Eppendorf tubes (200 µl). Lipids were extracted as previously described (Sarafian et al. 2014), iso-propanol (IPA) chilled at – 20 °C was added (600 µl) to the plasma. Samples were then vortex mixed for 1 min and incubated for 10 min at room temperature, then stored at – 20 °C overnight to improve protein precipitation. The following day, samples were centrifuged at 14,000×*g* for 20 min at room temperature. For each sample, the supernatant was transferred in correctly labelled LC vials. For quality control, a volume of 30 µl was taken from each sample, pooled in a tube and vortexed to create pooled quality control samples (QC). A volume of 100 µl of pooled samples were aliquoted in different QC vials.

### Global lipidomics profiling analysis

Samples were analysed using an ultra-high performance liquid chromatography quadrupole time-of-flight (UPLC-Q-TOF) mass spectrometry. The UPLC system was a Waters ACQUITY system (Waters Corp, Wilmslow, UK), coupled with a BEH C18, 1.7 µm, 2.1 × 100 mm analytical column (Waters Corp, Wexford, Ireland). The samples were analysed in a randomised order, and as technical triplicates, with an injection volume of 4 µl (ESI+ ) and 7 µl (ESI−). Pooled quality control samples (QC) were injected to condition the column (n = 8 injections) prior to the start of the analysis, and thereafter every tenth injection. A 23 min gradient elution was applied at a flow rate of 0.4 ml/min using two mobile phases, a mix of ACN and water (60:40, v:v) with 10 mM of ammonium formate (A), and a mix of IPA and ACN (90:10, v:v) with 10 mM of ammonium formate (B). The elution gradient was as follows: initial conditions at 30% B; from 1 to 15 min increased up to 99% B; from 15 to 20 min, maintained at 99% B; from 20 to 22 min decreased to 30% B; from 22 to 23 min, returned to initial conditions of 30% B. During the analysis, samples were maintained at 4 °C and the column at 65 °C Mass spectrometry analysis was performed using a Synapt G2-S Q-ToF (Waters Corp, Wilmslow, UK) with data collected in continuum format using positive and negative electrospray ionisation (ESI±). The data independent acquisition (DIA) mode, MS^e^ (Bateman et al. 2002; Silva et al. 2005) was used for data acquisition. Data were acquired from 50 to 1500 m/z range, in resolution mode. Precursor (low energy) and fragment (high energy) ion data were collected within the same acquisition with a scan time of 0.1 s for each, providing a total cycle time of 0.2 s. In the case of high energy, a linear collision energy ramp (20–40 eV) was applied over the 0.1 s scan. Capillary voltage was set to 1.5 kV, sampling cone to 30 V and extraction cone to 5 V. The source was set at 120 °C, and desolvation temperature at 650 °C. Desolvation gas flow rate was set at 800 l/h and cone gas at 50 l/h. Detector set up was performed using leucine enkephalin (LeuEnk) (Waters, Wexford, Ireland) and SYNAPT was calibrated using sodium formate (Waters, Wexford, Ireland), according to manufacturer’s instructions. Real time lock-mass correction was achieved by infusing LeuEnk at 10 μl/min through a lock-spray probe and acquired every 30 s.

### Data pre-processing

MS^e^ data were processed using Progenesis QI version 2.4 (Nonlinear dynamics, Newcastle, UK). Progenesis QI is a data analysis software package that accurately processes LC–MS data, based on accurate mass measurements, isotopic fit and fragmentation data. Data were chromatographically aligned using an appropriate pooled QC as the alignment reference. Data were peak picked and normalised to all compounds. Adducts corresponding with M + H, M + H-H_2_O, M + H − 2H_2_O, M + NH_4_, M + Na, M + K, M + 2H, M + 2Na were considered during peak picking. Compound measurements (raw and normalised compound abundances) were exported from Progenesis QI for downstream statistical analysis.

### Statistical analysis

For statistical analysis of the demographic and clinical data we applied the Student *T* test, Mann–Whitney U test, or Pearson χ^2^ test, and multiple comparisons corrections as appropriate (IBM SPSS Statistics 24). Results were considered statistically significant if the p value was less than 0.05 (Table 1). Randomisation of samples before preparation was performed using block randomisation based on the patients’ BMI and the outcome. No significant dependency between measurement order, the outcome and biometric and clinical information about patients was observed (p < 0.05; Mann–Whitney U test, Spearman correlation, Chi square test and Kruskal–Wallis test as applicable; Benjamini and Hochberg step-up FDR-controlling procedure) (Benjamini and Hochberg 1995).

Statistical analyses of UPLC-MS data were performed using the R statistical software (R Core Team 2013) and the Bioconductor package limma (Ritchie et al. 2015). UPLC-MS analyses were performed with two electrospray ionisation (ESI) modes (ESI+  and ESI−), which differ significantly in their analytical sensitivity and technical variability and therefore analysed independently. Briefly, median normalisation was performed on the raw UPLC-MS data to correct for the systematic measurement biases known to be associated with the UPLC-MS platform used. Quality control procedures were applied to check for systematic measurement biases (Broadhurst et al. 2018). Measurement precision was checked for each feature by computing the missing rate and coefficient of variation (CV) over the replicate measurements. Features with a missing rate greater or equal to 20% and features with a CV greater or equal to 30% were not considered in subsequent analyses. Features predictive for the outcome were selected using the empirical Bayes method, adjusting the design for replicate measures (Smyth 2004). Empirical Bayes methods have been successfully applied on LC–MS data (Kammers et al. 2015; Margolin et al. 2009), and offer the advantage of correcting for false discovery rate while identifying differential features. This approach was verified by comparing them to feature selection made using Mann–Whitney U test, on the average measurement per patient with multiple testing correction. Agreement between the two methods was demonstrated (Data not shown).

### Putative annotation of lipids

The exact mass of features with a significant adjusted value (p < 0.05) were searched against the Human Metabolome Database version 4.0 (Wishart et al. 2018), Lipid Maps version of January 2019 (Cotter et al. 2007) and Chemspider (searching the databases ChEBI version of February 2018, and KEGG version of April 2014; https://www.chemspider.com), using the Progenesis QI identification tool. Search parameters were set for an exact mass tolerance of 5 and 10 ppm, for precursor and fragment ions respectively. Putative annotations were then manually verified when fragmentation data was available, as shown in Supplementary Material 1. In accordance with the MSI reporting standards, we have achieved metabolite identification level 2, or putatively annotated compounds (Salek et al. 2013).

## Results

**Fig. 2:**
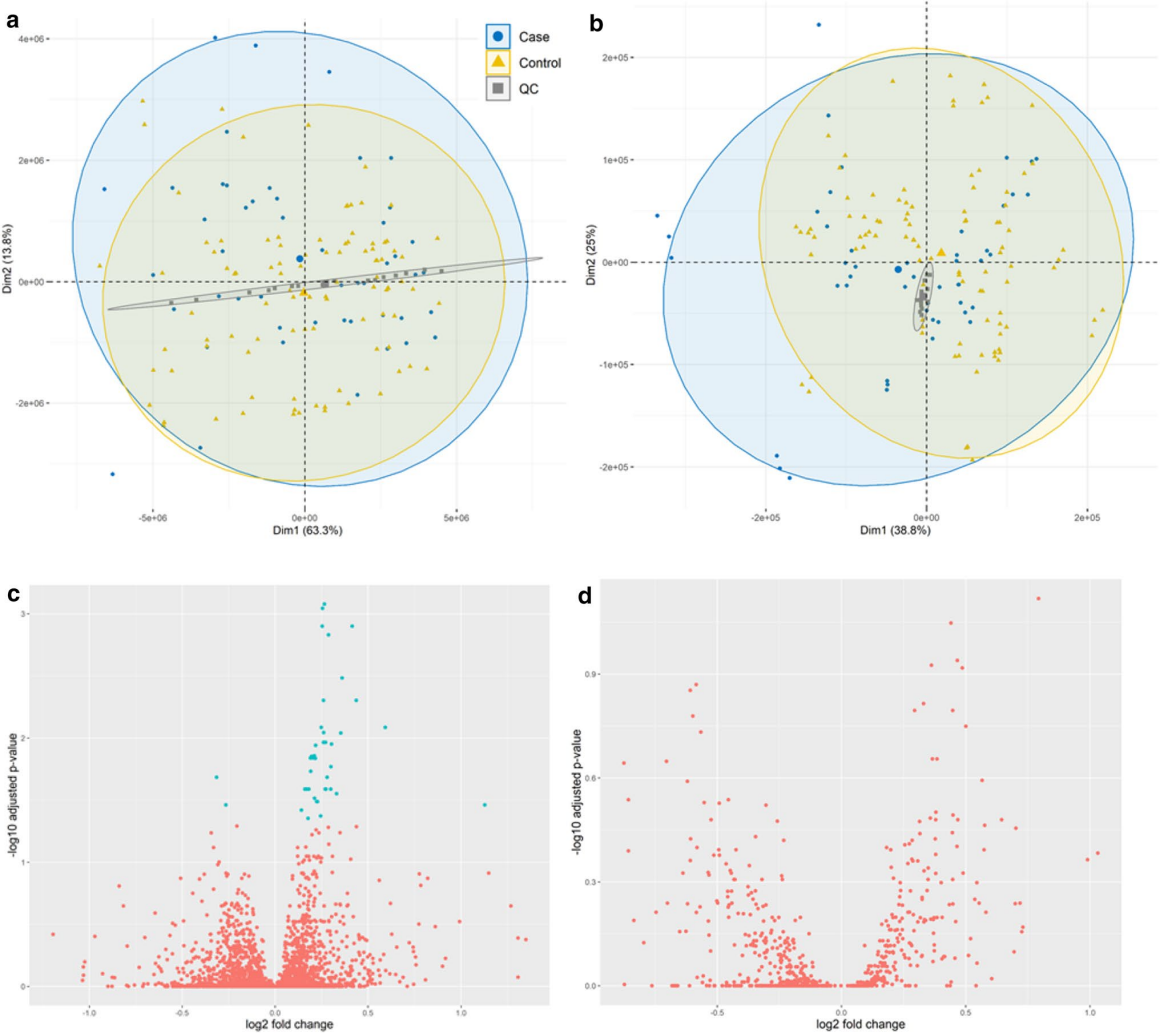
PCA score plot of all samples in ESI+  (**a**) and in ESI− (**b**) and respective volcano plots (**c** and **d**). In PCA plots, blue dots represent sPTB case samples, yellow triangles represent control samples, and grey squares represent QC samples. Volcano plots show log_2_ fold change plotted against –log_10_ of adjusted p values in ESI+  (**c**) and ESI − (**d**), plotted in blue are the variables with an adjusted p value < 0.05

We undertook lipidomics analysis of plasma samples from women participating in the SCOPE pregnancy cohort in Cork, Ireland. Samples were collected at 20 weeks’ gestation, and selected cases (delivery before 34 weeks’ gestation) were matched to controls according to age, BMI and ethnicity (Table 1). The clinical data analysis showed that women who had a spontaneous preterm birth were more likely to be married (43.5%), or single (37.5%), while only 18.8% were in a stable relationship. The ESI+  data set generated for analysis included 5878 features, and principal component analysis (PCA) and volcano plots are shown in Fig. 2a and c. After data pre-processing, data filtration (as described above), statistical analysis was performed using empirical Bayes methods on 4845 features. We putatively annotated 45 features as significantly differentially expressed between cases and controls, of which 27 remained significant following correction for multiple testing (Table 2). Of these 27 annotated lipids, 26 are phospholipids: 20 glycerophospholipids (12 phosphatidylcholines (PC), 7 phosphatidylethanolamines (PE), and 1 phosphatidylinositol (PI)), and 6 sphingolipids (2 ceramides and 4 sphingomyelins (SM)). Notably, all phospholipids were significantly lower in cases compared to controls. The last metabolite identified was a diglyceride, (DG (34:4)), detected at higher levels in sPTB compared to controls. Box plots of significant metabolites annotated in the Cork cohort are represented in Supplementary Material 2. The ESI-data set generated a total of 769 features, PCA and volcano plots are shown in Fig. 2b and d, and after data pre-processing, data filtration, and statistical analysis, no features were found to be significantly altered in sPTB group compared to control group.

**Table 2:** Putatively annotated lipids that were significantly (p < 0.05) altered in spontaneous preterm (sPTB) group compared to control

Identification	Chemical class	p value	Adjusted p value	Direction	Fold change (95% CI)
PC(35:1)	Glycerophospholipid	2.06E−05	**1.46E−02**	Down	1.23 (1.11–1.39)
PC(40:5)	Glycerophospholipid	1.30E−04	**2.43E−02**	Down	1.26 (1.10–1.45)
PC(42:4)	Glycerophospholipid	5.53E−08	**7.11E−04**	Down	1.19 (1.12–1.26)
PC(42:5)	Glycerophospholipid	2.75E−05	**1.21E−02**	DOWN	1.15 (1.07–1.27)
PC(O-38:4)	Glycerophospholipid	5.40E−06	**1.22E−02**	Down	1.16 (1.07–1.26)
PC(O-42:4)	Glycerophospholipid	7.51E−06	**7.51E−03**	Down	1.20 (1.09–1.26)
PC(O-44:6)	Glycerophospholipid	4.68E−05	**2.14E−02**	Down	1.13 (1.06–1.19)
PC(P-36:1)	Glycerophospholipid	8.61E−05	**2.73E−02**	Down	1.17 (1.07–1.26)
PC(P-38:3)	Glycerophospholipid	6.33E−05	**2.14E−02**	Down	1.13 (1.07–1.20)
PC(P-40:3)	Glycerophospholipid	8.19E−05	**2.14E−02**	Down	1.23 (1.14–1.36)
PC(P-40:4)	Glycerophospholipid	2.75E−05	**2.11E−02**	Down	1.12 (1.06–1.18)
PC(P-40:5)	Glycerophospholipid	2.79E−08	**6.57E−04**	Down	1.20 (1.12–1.29)
PE(38:0)	Glycerophospholipid	9.60E−06	**9.12E−03**	Down	1.21 (1.11–1.29)
PE(38:1)	Glycerophospholipid	3.26E−05	**3.69E−02**	Down	1.13 (1.06–1.21)
PE(38:2)	Glycerophospholipid	7.61E−05	**2.14E−02**	Down	1.21 (1.09–1.34)
PE(40:3)	Glycerophospholipid	1.31E−05	**9.56E−03**	Down	1.23 (1.12–1.35)
PE(40:4)	Glycerophospholipid	1.70E−05	**9.68E−03**	Down	1.16 (1.09–1.26)
PE(42:6)	Glycerophospholipid	9.97E−06	**7.12E−03**	Down	1.51 (1.27–1.78)
PE-Cer(38:3)	Glycerophospholipid	3.34E−06	**2.71E**−**02**	Down	1.17 (1.11–1.22)
PI(35:0)	Glycerophospholipid	4.83E−05	**2.99E**−**02**	Down	2.19 (1.53–3.17)
CerP(44:1)	Sphingolipid	7.17E−05	**2.14E**−**02**	Down	1.21 (1.10–1.30)
GalactosylCer(42:2)	Sphingolipid	2.48E−07	**1.07E**−**03**	Down	1.19 (1.12–1.27)
SM(32:2)	Sphingolipid	7.37E−05	**2.58E**−**02**	Down	1.16 (1.08–1.26)
SM(34:1)	Sphingolipid	1.25E−04	**4.63E**−**02**	Down	1.17 (1.09–1.25)
SM(35:1)	Sphingolipid	9.00E−07	**7.68E**−**03**	Down	1.28 (1.17–1.40)
SM(38:2)	Sphingolipid	9.68E−06	**1.21E**−**02**	Down	1.14 (1.08–1.20)
DG(34:4)	Fatty acyl	1.29E−04	**2.99E**−**02**	Up	1.20 (1.08–1.33)

Bold: adjusted p value < 0.05. Fold change reported with 95% confidence interval

*Cer* ceramide, *CerP* ceramide-1-phosphate, *DG* diglyceride, *PC* phosphatidylcholine, *PE* phosphatidylethanolamine, *PE-Cer* ceramide phosphoethanolamine, *PI* phosphatidylinositol, *SM* sphingomyeline

## Discussion and conclusions

**Fig. 3:**
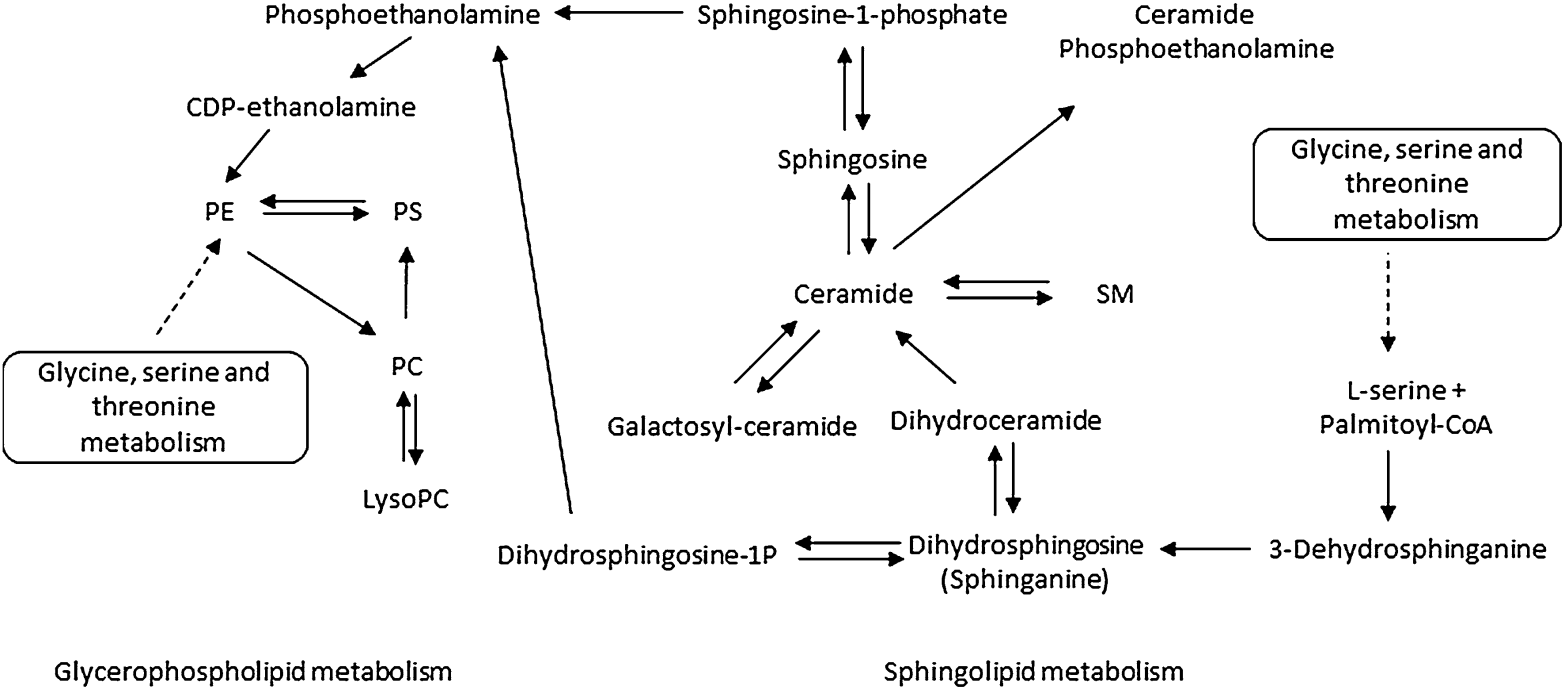
Simplified overview of Glycerophospholipid and Sphingolipid metabolisms. *PC* phosphatidylcholine, *PE* phosphatidylethanolamine, *PS* phosphatidylserine, *SM* sphingomyelin, *Palmitoyl-CoA* palmitoyl coenzyme A

Our study provides the first evidence that altered plasma phospholipids expression at 20 weeks’ gestation is associated with the onset of sPTB, and our findings are of broad relevance to “at risk” pregnancy states. Sphingolipid metabolism and glycerophospholipid metabolism are involved in pertinent molecular processes such as storage and breakdown of lipid molecules for energy, apoptosis, inflammation, and cell-membrane stabilisation (Baig et al. 2013). The sphingolipid and glycerophospholipid pathways are also interconnected and of mutual dependence, therefore it is plausible that an overall reduction in phospholipids are implicated in the pathophysiology of sPTB (Fig. 3). Alterations in phospholipid metabolism have previously been associated with pregnancy complications (Baig et al. 2013; Horgan et al. 2011) and animal models of pregnancy loss (Mizugishi et al. 2007), and our study represents a further step in understanding the involvement of phospholipid metabolism in sPTB. Future measures of altered phospholipid metabolism such as ELISA based technology, and integrated multi-omics techniques should be investigated—all of which hold promise for developing prognostic biomarkers for at risk pregnancy states.

Using a unique prospective cohort, we investigated blood plasma samples at 20 weeks’ gestation, from women whose pregnancies ended in sPTB (< 34 weeks of gestation) in comparison to matched controls, i.e. women who had uncomplicated pregnancies. We specifically putatively annotated 20 glycerophospholipids and 6 sphingolipids as differentially expressed following correction for multiple testing, and the uniform decreased expression of these two phospholipid groups was particularly striking in the sPTB group (see Fig. 2). In support of our observations in sPTB in SCOPE Cork, a recent metabolomic profiling study in the same cohort of women implicated bile acids, prostaglandins and Vitamin D as differentially expressed at 15 and 20 weeks’ gestation (Considine et al. 2019). This independent study which was performed at the University of Manchester, profiled metabolites using alternative extraction protocols (Dunn et al. 2011) and LC–MS/MS analyses. Despite different preparation methods and analytical analyses, both studies report increased fatty acids levels and reduced diacylglycerophosphoinositol in sPTB. Together, these studies provide complementary evidence for the implication of metabolomic and lipidomic pathways in the pathophysiology of sPTB and suggest dysregulation on a systems level.

In further support of altered lipid profiles in adverse pregnancy outcomes, Baig et al. (2013) reported altered glycerophospholipids and sphingolipids in placental syncytiotrophoblast microvesicles, in preeclampsia, which are shed from the placenta into maternal circulation (Baig et al. 2013). Evidence from animal models of at risk pregnancy also implicate altered lipid metabolism, whereby Mizugishi et al. (2007) reported that a critical enzyme of sphingolipid metabolism (sphingosine kinase, Sphk) was knocked out, resulting in early pregnancy loss in mice (Mizugishi et al. 2007). Finally, our findings of altered phospholipids in sPTB may be broadly related to the pathophysiology of obstetric antiphospholipid syndrome (OAPS), an auto-immune disease consistently associated with pregnancy complications. OAPS is characterised by presence of circulating antiphospholipid antibodies (aPL) (Antovic et al. 2018). These aPLs target a circulating blood protein, β_2_-glycoprotein I (β_2_-GPI), that has high affinity and binds to phospholipids present on the surface of cell membranes, and in doing so create a cascade of events that can lead to thrombosis, vasculopathy, inflammation, and a range pregnancy complications (Garcia and Erkan 2018). A recent systematic review aiming to examine all biomarkers of sPTB in human studies, from 1965 to 2008 (Menon et al. 2011), showed that 55% of the biomarkers studied are involved with inflammation and immune response, and that interleukin 6 (IL6), IL8 and corticotrophin-releasing hormone (CRH) were the most investigated biomarkers. The other biomarkers studied were involved in several other pathways, such as stress, hormonal metabolism, cellular metabolism, apoptosis, and placental dysfunction. In addition, proteins are the most studied type of biomarker in the articles included in this systematic review. The authors noted that none of these biomarkers showed the ability to predict sPTB accurately or help in better understanding the pathophysiology of this pregnancy adverse outcome. This shows how metabolomics based approaches could be better suited as biomarkers, since metabolites are a direct reflection of the cellular activity (Patti et al. 2012).

Our study has limitations, in particular the low number of sPTB samples with a delivery before 34 weeks of gestation, which is attributed to the fact that the participants recruited for SCOPE were low-risk nulliparous women. Nevertheless, our study was well designed with a 1:2 case:control ratio, participants were carefully matched for BMI, age and ethnicity, and we applied multiple testing to identify 27 significant features following multiple testing correction. Moreover, no additional findings were determined from the remaining metadata. Overall, our findings warrant replication in larger independent cohorts, using multi-omics measures to confirm dysregulation on a systems level, and explore whether the contribution of these 27 altered lipids is a cause or effect of preterm birth.

In conclusion, this study reports reduced glycerophospholipids and sphingolipids at 20 weeks’ gestation, prior to the onset of sPTB in women in the Cork SCOPE study. Our findings confirm and extend findings by Considine et al. (2019), suggesting convergent metabolomic and lipidomic pathways in the same cohort of women (Considine et al. 2019), which may serve as potential therapeutic targets or putative markers for the prediction of sPTB. Further research is needed to validate these findings in independent pregnancy cohorts.

## Electronic supplementary material

Below is the link to the electronic supplementary material.

Supplementary file1 (DOCX 464 kb)

Supplementary file2 (DOCX 435 kb)
