# Supplementary material for: Association between phospholipid metabolism in plasma and spontaneous preterm birth: a discovery lipidomic analysis in the cork pregnancy cohort
Source: Metabolomics. 2020 Jan 24;16(2):19. doi: 10.1007/s11306-020-1639-6 (PMC6978438; doi:10.1007/s11306-020-1639-6)
Supplement: Supplementary file 1 — Supplementary file1 (DOCX 464 kb) [file 11306_2020_1639_MOESM1_ESM.docx]

Association between Phospholipid Metabolism in Plasma and Spontaneous Preterm Birth: A Discovery Lipidomics Analysis in the SCOPE Pregnancy Cohort.

Metabolomics Journal

Aude-Claire Morillon; Shirish Yakkundi; Gregoire Thomas; Lee A Gethings ; James I Langridge; Philip N Baker; Louise C Kenny, Jane A English; Fergus P McCarthy

Corresponding author: Jane A English, Department of Anatomy and Neuroscience, Western Gateway Building, Western Road, University College Cork, Cork, Ireland. Email: jane.english@ucc.ie


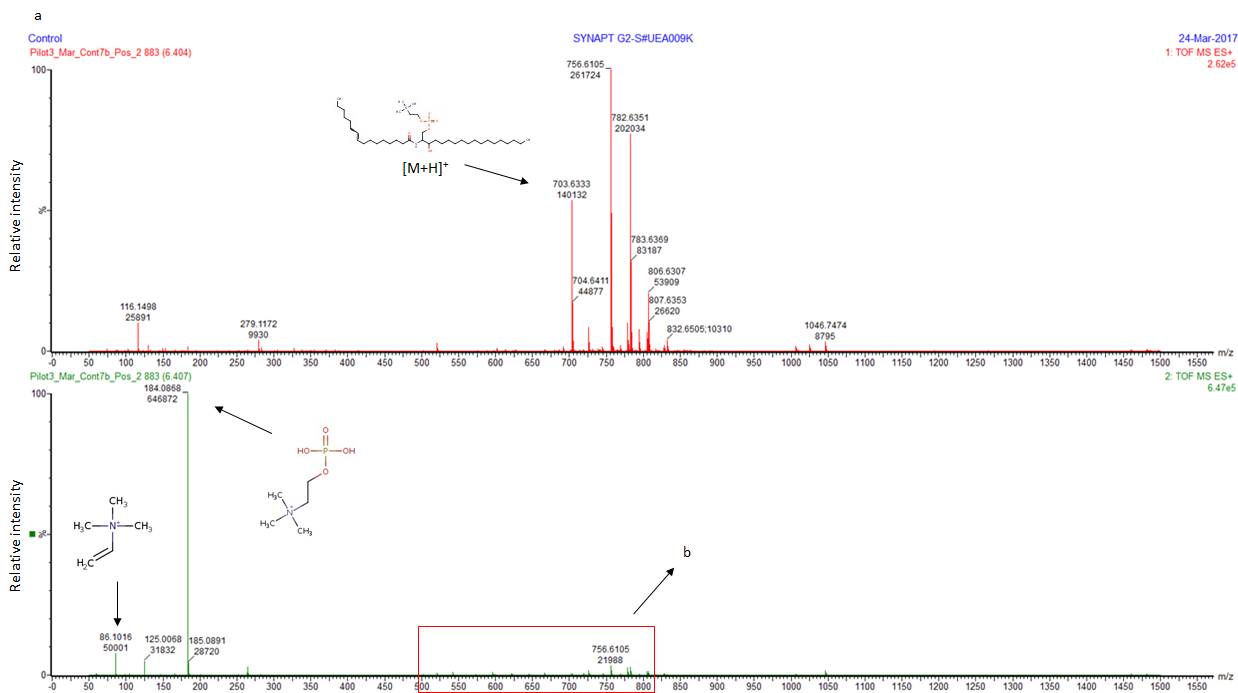


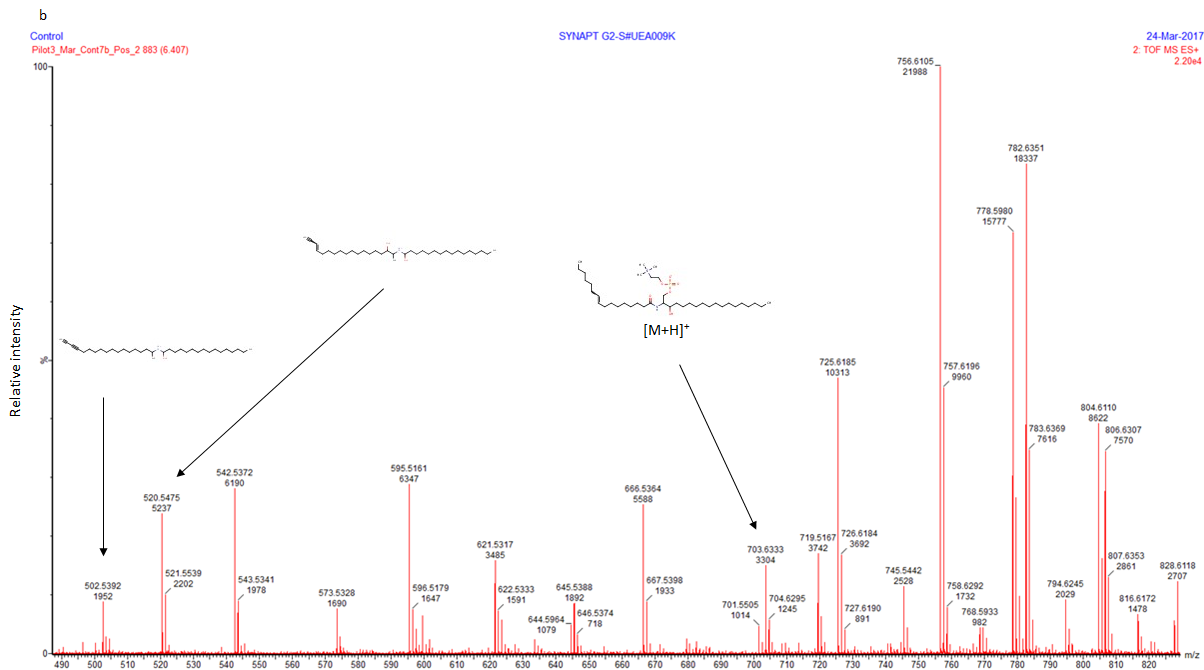


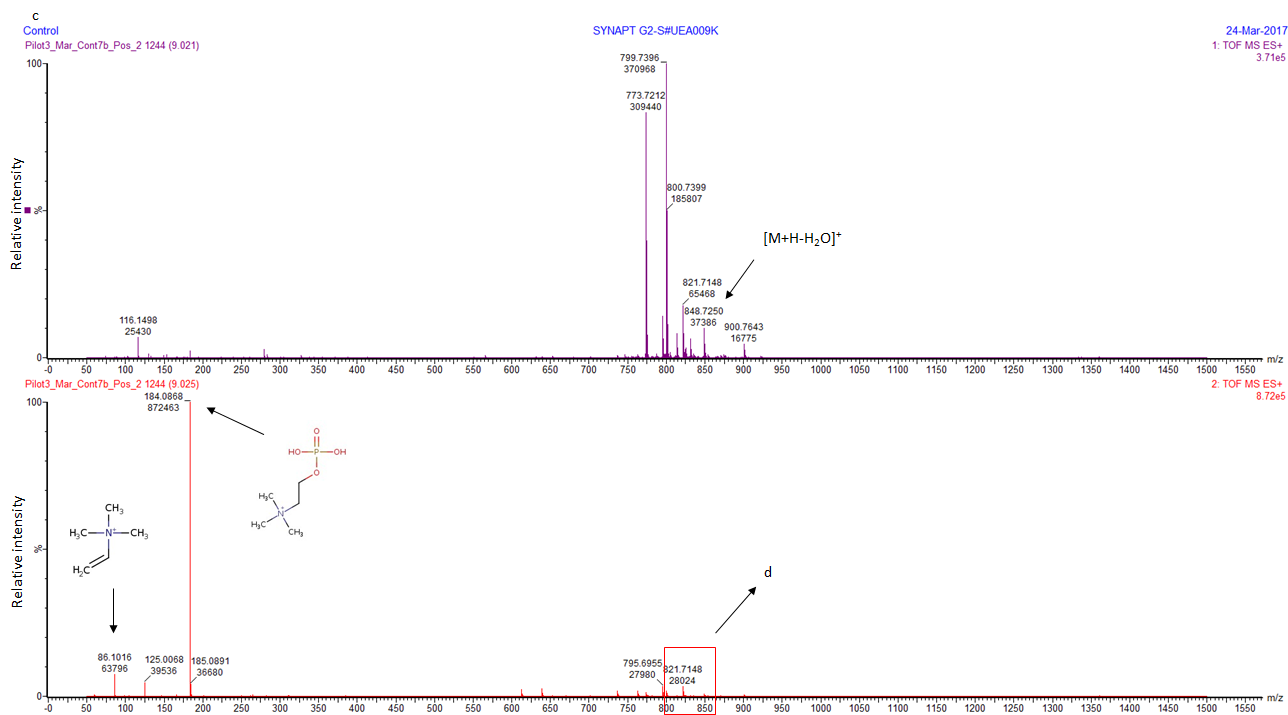


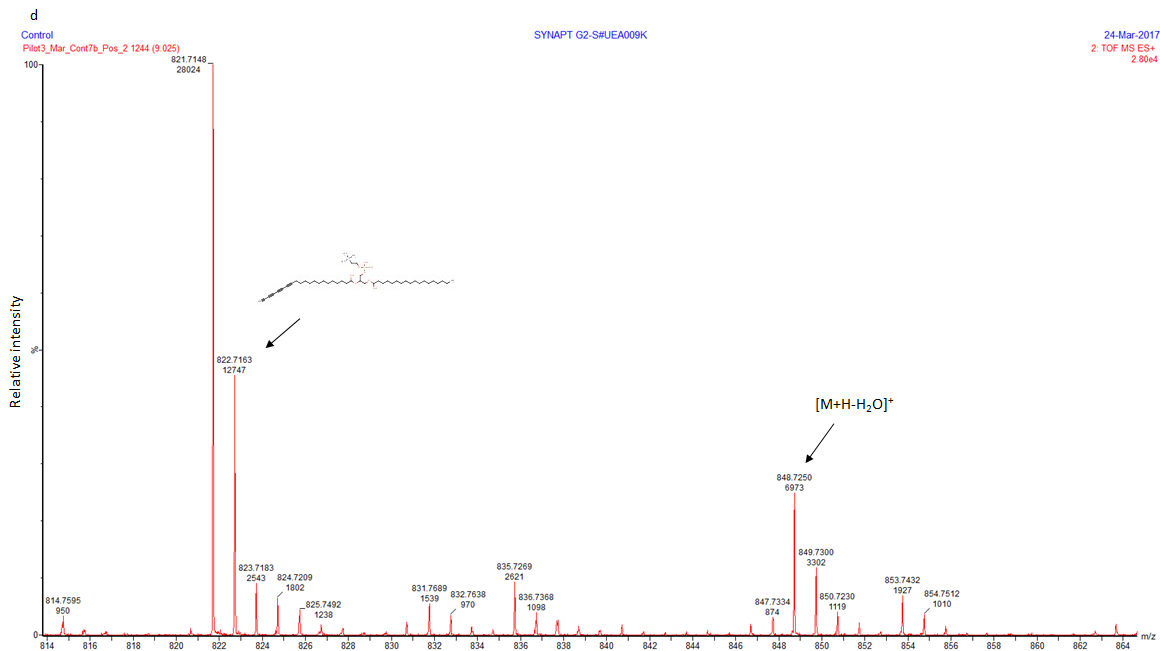


**electronic supplementary Fig. 1** Example of manual validation of two lipids

a: MS1 (top) and MS2 (bottom) of sphingomyelin SM(34:1) (HMDB0013464); b: MS2 of SM(34:1) zoom in range 490-830 m/z; c: MS1 (top) and MS2 (bottom) of phosphatidylcholine PC(42:4) (HMDB0008224); d: MS2 of PC(42:4) zoom in range 814-865 m/z
