# Supplementary material for: Association between phospholipid metabolism in plasma and spontaneous preterm birth: a discovery lipidomic analysis in the cork pregnancy cohort
Source: Metabolomics. 2020 Jan 24;16(2):19. doi: 10.1007/s11306-020-1639-6 (PMC6978438; doi:10.1007/s11306-020-1639-6)
Supplement: Supplementary file 2 — Supplementary file2 (DOCX 435 kb) [file 11306_2020_1639_MOESM2_ESM.docx]

Association between Phospholipid Metabolism in Plasma and Spontaneous Preterm Birth: A Discovery Lipidomics Analysis in the SCOPE Pregnancy Cohort.

Metabolomics Journal

Aude-Claire Morillon; Shirish Yakkundi; Gregoire Thomas; Lee A Gethings; James I Langridge; Philip N Baker; Louise C Kenny, Jane A English; Fergus P McCarthy

Corresponding author: Jane A English, Department of Anatomy and Neuroscience, Western Gateway Building, Western Road, University College Cork, Cork, Ireland. Email: jane.english@ucc.ie


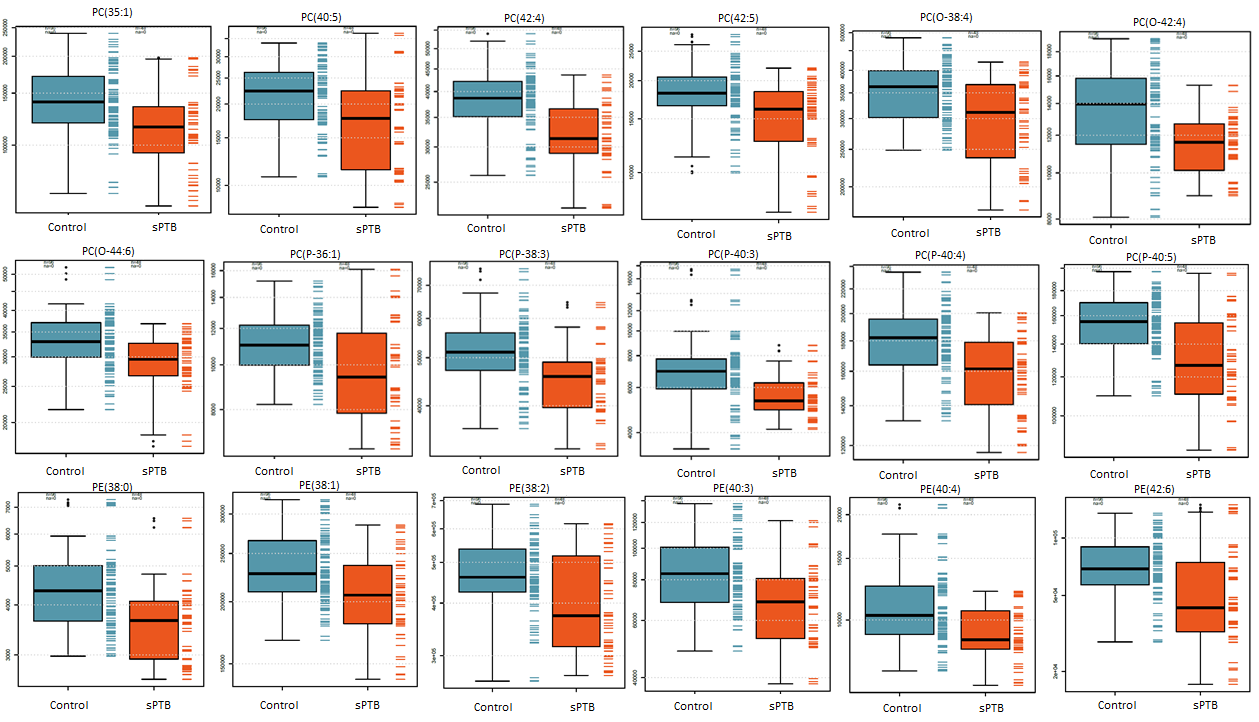


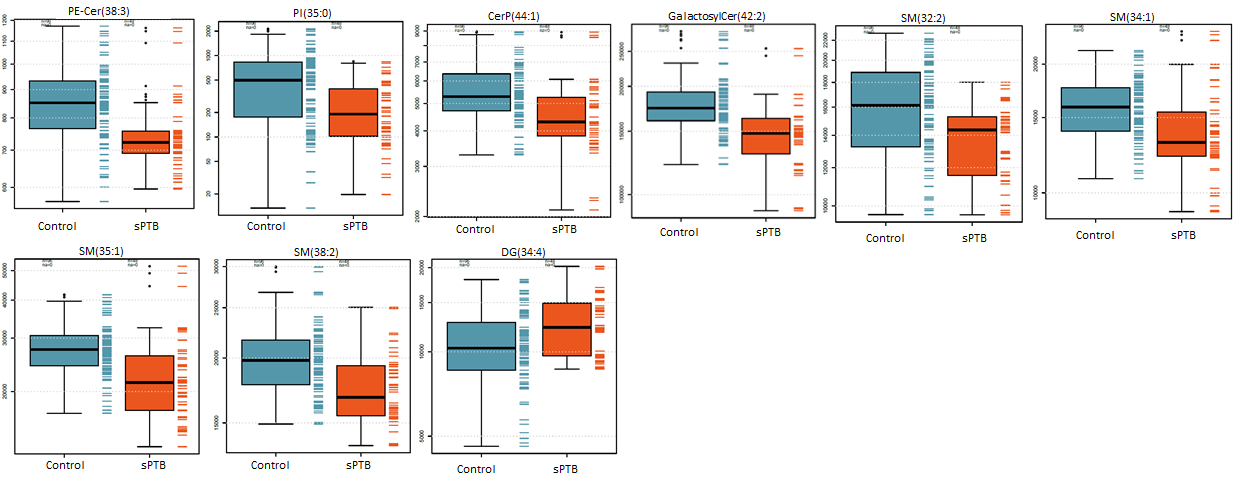


**electronic supplementary Fig.2** Box plots showing the normalised intensity of features significantly altered (adjusted p-value < 0.05) in spontaneous preterm birth (sPTB) group (orange box) compared to control group (blue box), from semi-targeted UPLC-MS analysis of SCOPE Cork (cases n=16, controls n=32). Segments represent observations from all triplicate injections. In each box, the solid black line represents the median, the top of the box is the third quartile (Q3) and the bottom is the first quartile (Q1). The length of the box (Q1 to Q3) shows the interquartile range (IQR). The bottom whisker shows the lowest value observed or Q1-1.5xIQR, depending on which is bigger. The top whisker shows the highest value observed, or Q3+1.5xIQR, depending on which is smaller
